# Natural Killer Cell Dependent Within-Host Competition Arises during Multiple MCMV Infection: Consequences for Viral Transmission and Evolution

**DOI:** 10.1371/journal.ppat.1003111

**Published:** 2013-01-03

**Authors:** Andrea R. McWhorter, Lee M. Smith, Laura L. Masters, Baca Chan, Geoffrey R. Shellam, Alec J. Redwood

**Affiliations:** Microbiology and Immunology, School of Pathology and Laboratory Medicine, M502, University of Western Australia, Crawley, Western Australia, Australia; McMaster University, Canada

## Abstract

It is becoming increasingly clear that many diseases are the result of infection from multiple genetically distinct strains of a pathogen. Such multi-strain infections have the capacity to alter both disease and pathogen dynamics. Infection with multiple strains of human cytomegalovirus (HCMV) is common and has been linked to enhanced disease. Suggestions that disease enhancement in multi-strain infected patients is due to complementation have been supported by trans-complementation studies in mice during co-infection of wild type and gene knockout strains of murine CMV (MCMV). Complementation between naturally circulating strains of CMV has, however, not been assessed. In addition, many models of multi-strain infection predict that co-infecting strains will compete with each other and that this competition may contribute to selective transmission of more virulent pathogen strains. To assess the outcome of multi-strain infection, C57BL/6 mice were infected with up to four naturally circulating strains of MCMV. In this study, profound within-host competition was observed between co-infecting strains of MCMV. This competition was MCMV strain specific and resulted in the complete exclusion of certain strains of MCMV from the salivary glands of multi-strain infected mice. Competition was dependent on Ly49H^+^ natural killer (NK) cells as well as the expression of the ligand for Ly49H, the MCMV encoded product, m157. Strains of MCMV which expressed an m157 gene product capable of ligating Ly49H were outcompeted by strains of MCMV expressing variant m157 genes. Importantly, within-host competition prevented the shedding of the less virulent strains of MCMV, those recognized by Ly49H, into the saliva of multi-strain infected mice. These data demonstrate that NK cells have the strain specific recognition capacity required to meditate within-host competition between strains of MCMV. Furthermore, this within-host competition has the capacity to shape the dynamics of viral shedding and potentially select for the transmission of more virulent virus strains.

## Author Summary

Infection of the host with multiple strains of a pathogen is common and occurs with the herpesvirus, human cytomegalovirus (HCMV). However the effects of multi-strain infection on the host and the pathogen remain poorly studied. Here we show, in a mouse model, that infection of C57BL/6 mice with multiple strains of murine CMV (MCMV) results in profound within-host competition. Competition between the strains of MCMV is dependent on Ly49H^+^ natural killer (NK) cells. The NK cell activation receptor Ly49H receptor targets certain genotypes of the viral protein, m157. During multi-strain infection, strains of MCMV encoding an m157 capable of binding Ly49H are excluded from the salivary gland and the saliva of C57BL/6 mice, allowing for the shedding of only non-Ly49H binding strains of MCMV in the saliva. This within-host competition could therefore have significant impacts on the circulation of MCMV strains, as only the most virulent MCMV strains were present in the saliva.

## Introduction

It is becoming increasingly clear that many infections are caused by multiple distinct strains of the infecting pathogen. A recent review documented 51 infections of humans in which there is definitive evidence of such multi-strain infection [1]. This is likely to be an underestimation of the true rate of multi-strain infection, given the technical difficulties associated with the detection of more than one pathogen strain. Multi-strain infection has been reported with many pathogen species including bacteria, protozoa, helminths, fungi and viruses. In humans, multi-strain infection has been demonstrated for a number of viruses including; HIV, dengue virus, papillomavirus, hepatitis B, C, D and E viruses and rotavirus (reviewed in [1]). Multi-strain infection appears to be particularly common among the herpesviruses and has been demonstrated for herpes simplex virus types 1 [2] and 2 [3], Epstein Barr virus [4], varicella-zoster virus [5], human herpesvirus 8 [6] and human cytomegalovirus (HCMV) [7], [8].

HCMV, a member of the betaherpesvirus subfamily, is a large double stranded DNA virus with a worldwide prevalence of 55–100% depending on socioeconomic status and geographical location. HCMV infection is life-long but is generally asymptomatic in the immunocompetent host. However in the immunocompromised individual HCMV can cause significant morbidity and mortality. Despite the advent of antiretroviral therapy, AIDS patients remain at risk of HCMV induced retinitis, uveitis and vitritis [9]. HCMV has become the most common cause of intrauterine viral infection in industrialized nations and causes congenital abnormalities such as sensorineural hearing loss and mental retardation [10]. For solid organ and bone marrow allograft recipients, HCMV remains a major opportunistic pathogen and causes post transplant complications including vascular stenosis, CMV disease and organ rejection [11].

Infection with multiple strains of HCMV was first reported in immunocompromised, HIV infected, individuals more than 25 years ago [7], [8]. Since then a series of studies have documented multiple HCMV infection in the immuno-suppressed or -compromised including; patients with leukemia and lymphoma [12], [13], solid organ allograft recipients [14] and bone marrow recipients [15]. Donor tissues including the kidneys [16], [17] the heart [18], the liver [19] and the lungs [20] have all been shown to transmit virus capable of re-infecting seropositive transplant recipients. In the immunosuppressed, rates of multi-strain infection can be high. In HIV infected patients, multi-strain HCMV infection can be as high as 40–46% [21], [22]. Even higher rates of multi-strain infection have been documented in allograft transplant recipients, with one study of lung transplant recipients identifying multi-strain infection in 90% of patients [20]. The number of individual infecting strains can also be high with up to 8 individual strains of HCMV detected in a single patient [23].

Multi-strain HCMV infection can also be found in normal healthy individuals [24], [25], indicating that neither immunosuppression nor transplant-acquired re-infection is needed for the acquisition of multiple HCMV strains. In children attending day care centers the rate of multiple infection can be as high as 17%, with up to 6 different strains detected in sequential sampling studies [26]. Four of eight women attending an STD clinic were infected with multiple strains of HCMV [25], while studies of tissues obtained from the necropsy of 25 immunocompetent individuals identified multi-strain infection rates of 20% [27]. Using strain specific serology testing Ross and colleagues demonstrated re-infection rates of 29% in a cohort of 205 seropositive women [28]. In regions or socioeconomic groups with high rates of seroprevalence, multi-strain infection has been demonstrated in the majority of infected individuals [29], [30].

Theoretical and empirical studies suggest individual strains of pathogen can participate in either positive (complementary) or negative (competitive) interactions when co-infecting the same host. These interactions can affect a range of medically relevant outcomes such as: strain selection post vaccination, the spread of drug resistance, genetic exchange and the evolution of virulence (reviewed in [1]). Given the frequency with which individuals are infected with multiple strains of HCMV, a full understanding of the effects which multi-strain infection has on the pathobiology of infection is required to fully understand treatment options, control measures and diseases processes. For instance, complementary interactions between viral strains have the obvious potential to enhance disease severity. Several studies of immunosuppressed allograft transplant recipients have linked multi-strain HCMV infection to enhanced disease, elevated viral loads and reduced organ survival [13], [17], [19], [20], [31], [32]. Similarly, multi-strain infection in immunocompromised patients has also been linked to enhanced CMV disease [13] or even accelerated progression to AIDS in HIV infected individuals [21]. Primary infection reduces, but does not protect from congenital disease, with morbidity seen in infants born to mothers who were seropositive prior to pregnancy (reviewed in [33]). While this could be due to viral re-activation from latency, reinfection with novel strains of HCMV can cause congenital disease [34], [35] and has been linked to enhanced fetal mortality [36].

Synergistic interaction amongst co-infecting strains of HCMV may be a cogent explanation for the enhanced disease seen in patients infected with multiple strains. However, not all studies have demonstrated enhanced disease or viral loads in multi-strain infected individuals. Complementation between co-infecting strains of CMV has only been demonstrated in the mouse model between gene knockout viruses and wild type strains of murine CMV (MCMV) [37]–[39]. Complementation between naturally circulating strains of CMV has not been assessed. Finally, it is not clear that complementation, rather than competition or even neutrality, will be the natural outcome of co-infection with multiple strains of CMV. Indeed many theoretical models predict that competition between strains of co-infecting pathogens is a likely outcome of multi-strain infection, and perhaps more importantly that such competition can lead to the evolution of enhanced virulence (reviewed in [40]).

In order to investigate empirically the outcome of multi-strain CMV infection we have developed a novel mouse model using naturally circulating strains of MCMV. As with HCMV in humans, multi-strain MCMV infection of free-living mice is common [41], [42]. The C57BL/6 (B6) mice used in this study show resistance or susceptibility to MCMV infection based on the expression of a single MCMV gene product, m157. B6 mice express the NK cell activation receptor Ly49H, which recognizes virally infected cells [43]–[45] via direct recognition of m157 [46], [47]. However, strains of MCMV express different genotypes of m157 [48], [49], most of which are not recognized by Ly49H [50]. B6 mice are susceptible to infection with m157^Ly49H−^ strains of MCMV but resistant to m157^Ly49H+^ strains [48], [49]. The wild-derived viruses used in the present study were all isolated from a single wild-caught mouse [42] and are distinct strains as determined by full genome sequencing [49], [51].

We noted no evidence of within-host complementation, with no enhancement of viral titers in mice receiving the mixed inoculum compared to mice receiving a single strain infection. In marked contrast profound, MCMV strain specific, within-host competition was observed in B6 mice. During single strain infection all four viral strains, C4A, C4B, C4C and C4D disseminated to and replicated within the salivary glands of mice. However, during multi-strain infection, both C4A and C4B were excluded from the salivary glands, while C4C and C4D replicated normally at this site. Functional studies demonstrated that the m157 protein produced by both C4A and C4B ligated Ly49H, whereas C4C and C4D m157 proteins failed to ligate this receptor. Subsequent studies using Ly49H congenic mice, Ly49H blocking antibodies and NK cell depletion studies demonstrated that within-host competition was dependent on Ly49H^+^ NK cells. Competition was also noted between K181 (m157^Ly49H+^) and C4C (m157^Ly49H−^), but not between K181^Δm157^ (m157^Ly49Hnull^) and C4C (m157^Ly49H−^), confirming a requirement for an m157 protein capable of binding Ly49H in the competition described here. Within-host competition was complete and prevented the shedding of m157^Ly49H+^ strains of MCMV into the saliva during co-infection with m157^Ly49H−^ strains. Within-host competition was evident as early as day three p.i. suggesting that NK cells prevented the dissemination of m157^Ly49H+^ to the salivary glands. Therefore these studies demonstrate that NK cells are capable of mediating competition between strains of MCMV and have the potential to shape dissemination patterns of this virus.

## Materials and Methods

### Ethics statement

Experiments were performed with the approval of the Animal Ethics and Experimentation Committee at the University of Western Australia and in accordance with the guidelines of the National Health and Medical Council of Australia.

### Mice

Highly inbred, female BALB/cArc (BALB/c) and C57BL/6JArc (B6) mice were obtained from the Animal Resource Centre (Perth, Western Australia). The congenic mouse strains, BALB.B6-*Cmv1^r^* and B6.BALB.TC1 (kindly provided by Dr. Anthony Scalzo, Lions Eye Institute, University of Western Australia), were bred at the Animal Services Facility, at the University of Western Australia. All mice were infected at seven to eight weeks of age and were maintained under specific pathogen free conditions prior to and during experimentation.

### Cells and cell lines

Primary mouse embryonic fibroblasts (MEF) were grown in minimal essential media (MEM, HyClone, Thermo Scientific, Logan, UT) supplemented with 8% newborn calf serum (NCS, HyClone) and antibiotics (1 unit penicillin and 1 µg streptomycin/ml (Invitrogen, Sydney)). The BWZ-HD12 reporter cells (kindly provided by Dr. Anthony Scalzo) were grown in RPMI-1640 (Invitrogen) supplemented with 10% fetal bovine serum (FBS, HyClone), antibiotics, 2 mM glutamine, 1 mM pyruvate, 50 µM 2-mercaptoethanol and 200 µg/ml hygromycin. Reporter assays to measure the Ly49H binding capacity of individual MCMV strains were performed as previously described [46]. Briefly, 5×10^3^ BALB/c derived MEF were added to each well of a flat bottom 96 well tray in 1×MEM supplemented with 2% NCS plus antibiotics and incubated overnight at 37°C in 5% CO_2_. MEF were infected with MCMV at a multiplicity of infection (MOI) of 0.5 by centrifugal enhancement. BWZ-HD12 reporter cells were added to infected MEFs 24–48 hours later when cytopathic effect in the infected MEF approached 100%. Infected MEF and reporter cells were incubated overnight at 37°C in 5% CO_2_, with the β-galactosidase production assessed eight hours after the addition of 0.15 M chlorophenol red-ß-D-galactopyranoside (CPRG). Chlorophenol red production was measured at a wavelength of 570 nm, with 630 nm used as a reference wavelength. Positive controls were 0.05 µg/ml PMA and 0.5 µg/ml ionomycin. K181 infected MEF were used as an additional positive control, and uninfected MEF served as a negative control.

The capacity of the MCMV strains used in this study to replicate in MEF was assessed by multi-step growth curves analysis as previously described [52]. The level of m157 on infected cells was assessed by infecting MEF at a high MOI (>10) with C4A, C4B, C4C, C4D or K181. MEF were stained 26 hours later with anti-m157 antibody (6H121, provided by Wayne Yokoyama, Washington University, School of Medicine) [53]. 6H121 conjugated to AlexaFluor647 was a kind gift of Dr Jerome Courdert, (Lions Eye Institute, Western Australia). Levels of m157 on infected MEF were assessed by gating on live cells (LIVE/DEAD Fixable Near-IR Dead Cell Stain, Molecular Probes) and were compared to uninfected cells.

### Viruses and infection protocols

The MCMV strains C4A, C4B, C4C, and C4D were isolated from a single free-living wild mouse, *Mus musculus domesticus*, collected from a location near (C)anberra, Australian Capital Territory, Australia and have been previously described [42], [49]. Entire genome sequencing identified these viruses as distinct strains of MCMV (Accession numbers; EU579861, HE610452, HE610453, HE610456). K181^Perth^ (hereafter referred to as K181) has been described previously [54]. Viruses were propagated *in vitro* on BALB/c MEF and were maintained as low passage stocks.

All *in vivo* experiments were performed with secondary salivary gland derived virus stocks. These stocks were raised by two passages of virus in weanling (three week old) female, B6 mice for inoculation of B6 background mice, or in BALB/c mice for inoculation of BALB/c background mice. Control, single strain infected mice, were inoculated with 1×10^4^ pfu of virus via the intraperitoneal (i.p.) route. Multi-strain infections were also given via the i.p. route and comprised a mixed inoculum of up to four different strains of MCMV, with each strain being an equal fraction of the total 1×10^4^ pfu inoculum.

### Construction of K181^Δm157^

K181^Δm157^ was produced by recreating the 104 bp spontaneous loss-of-function mutation in *m157* found upon serial *in vivo* passage of K181 through Ly49H^+^ congenic mice [48]. Mutagenesis of K181 to produce K181^Δm157^ was performed by ET recombination as described [55]. For this we used the PCR primers; delta m157 forward TCTGGGACACACAAAGGATCTGCGTACGATATGCATGTCTGTTTTTGGGAACGTCGTGGAATGCCTTCGAATTC and delta m157 reverse CCCGAACGTTGCTTTTTATCATATAGGTACCAATTTCTTTTGCGTCGGAAACAAGGACGACGACGACAAGTAA . PCR primers contain sequences specific for the plasmid pFLRTKn (underlined) and sequence homologous to nt 216554–216603 and 216400–216449, respectively in K181 (accession number AM886412). The K181 bacterial artificial chromosome (BAC), pARK25, used for mutagenesis has been described [56]. The kanamycin resistance gene used to select for successful recombinants was removed by site-specific recombination using FLP recombinase expressed by the plasmid pCP20 [55]. Successful deletion of the 104 bp of *m157* in K181^Δm157^ was confirmed by sequence analysis (data not shown). K181^Δm157^ replicated like wild type virus *in vitro* and failed to stimulate the Ly49H reporter cell line BWZ-HD12 (data not shown).

### Virus determination in vivo

Viral titers in mice were determined by standard plaque assay [57]. Briefly, organs were weighed and combined with 0.1 mg/ml of 1×MEM, supplemented with 2% NCS in a 2.0 ml safe lock microcentrifuge tube with a 5 mm stainless steel bead. Organs were homogenized using a Qiagen TissueLyser II set at a frequency of 25 Hz for 2.5 minutes. Organ homogenates were clarified by centrifugation at 425 rcf. Dilutions of homogenates were added to confluent monolayers of MEF in 24 well tissue culture plates. Virus and cells were co-incubated for 1 hour at 37°C and the virus was removed and the cells overlaid with 1 ml of methylcellulose/1×MEM supplemented with 2% NCS and antibiotics. Plates were incubated for five days at 37°C in 5% CO_2_ to allow for plaque formation. Plaque assays were stopped after five days with addition of methylene blue stain containing 5% formaldehyde. After 24 hours the plates were washed, air-dried and plaques counted by light microscopy, all titers are expressed as plaque forming units (pfu) per gram of tissue.

Viral genomes in the tissues of infected mice were quantified by multiplex PCR. DNA was extracted from 10 mg of spleen, liver or salivary gland using the Wizard Genomic DNA kit according to the manufacturer specifications (Promega, Madison, Wisconsin). Primers, forward (5′-ACAGGACGACCGAGTTCCCCG-3′) and reverse (5′-GTTGYACATCTAAGATCGAGAAACA-3′) (GeneWorks, Adelaide, South Australia) were used to amplify the MCMV gene, *m144*. Strain-specific oligonucleotide probes were: C4A (FAM-ATTCGAACCTGTTCACTGGCG-BHQ1), C4B (HEX-CTTAGCTTCTTTTGACAGTCACGGTC-BHQ1) (both obtained from GeneWorks), C4C (LC610-CAAAGCGGCGCAGCAACATAAC-BHQ1), C4D (LC670-TCCAAACCCCAAACCAAAACCACGC-BHQ1) (Sigma-Aldrich, Castle Hill, New South Wales) and K181 (FAM-CCTCAAACGTCAAAACAACACCACGA-BHQ1) (GeneWorks). Reaction mix comprised 10 µl Roche (Basel, Switzerland) 2×Probes Master Mix and 500 nM primers. Probes were used at a concentration of 250 nM for C4A, K181, C4C and C4D and at 375 nM for C4B. A total of 100 ng genomic DNA was added to each reaction mix. Cycling conditions were as follows: 10 minutes 95°C, followed by 45 cycles each consisting of 30 seconds at 95°C and 2 minutes at 66°C, and a final cooling step to 50°C for 10 seconds. Concentrations of viral DNA were calculated relative to an internal standard control using the fit-points method on the LC480 software.

### In vivo antibody treatments

To deplete NK cells, mice were treated at day −1, 0 and then every five days with 150 µg of the antibody PK136 (anti-NK1.1). On day 0, mice were co-infected with 0.5×10^3^ pfu C4A plus 0.5×10^3^ pfu C4C of salivary gland derived virus stock. Control mice co-infected with C4A and C4C were treated with vehicle (PBS) alone. Efficacy of NK cell depletion was assessed at day 18 post infection (p.i.) by flow cytometry of splenocytes using the pan NK cell marker CD49b (clone DX5; BD Pharmingen, North Ryde, NSW). PK136 treatment resulted in a 38% reduction in NK cells relative to PBS treated mice. Ly49H blocking studies were performed with the antibody 3D10 (anti-Ly49H), which was provided by Professor Wayne Yokoyama (Washington University). Mice were injected with 200 µg of 3D10 on day −2 and day 0 and then every four days. On day 0 mice were infected as above with a mixed inoculum of C4A and C4C. Control mice co-infected with C4A and C4C were treated with isotype control antibody, 9E10 (anti-cMyc). All antibodies were supplied by WAIMR monoclonal antibody facility (AbSolutions, WAIMR, Perth WA) as a protein G affinity purified product.

### Collection of saliva samples

Shedding of virus into the saliva was measured by collecting saliva onto FTA paper (Qiagen, Doncaster, Victoria) [58]. A 1 mm^2^ punch was collected from air-dried, saliva impregnated paper. The paper punch was then washed in 100 mM Tris, 0.1% SDS for 30 minutes, then 5 M guanidine thiocyanate for 10 minutes, followed by three washes in sterile water for 10 minutes with a final 10 minute wash in 70% ethanol. Punches were air dried and then loaded directly into PCR wells for analysis by multiplex qPCR.

### Statistics

One way analysis of variance (ANOVA) followed by Tukey's comparison was used to determine statistical significance between multiple groups. Unpaired two tailed T tests were used to compare between data from two groups. All statistical analyses were performed using GraphPad Prism 5.0 (GraphPad Software, Inc, California) with the values are expressed as mean ± SEM.

## Results

### Multiple MCMV infection leads to competition in C57BL/6 mice

This study was designed to explore two potential consequences of multi-strain CMV infection. These were, inter-strain complementation leading to enhanced viral loads in the tissues of infected mice and within-host interactions, either complementary or competitive, that affected the replication capacity of individual strains. These outcomes were assessed by plaque assay and by measuring strain prevalence using strain specific quantitative PCR, respectively in B6 mice infected with up to four strains of MCMV. The strains used, C4A, C4B, C4C and C4D were all isolated from a single wild caught mouse and have been confirmed as distinct strains by full genome sequence analysis [49], [51].

**Figure 1:**
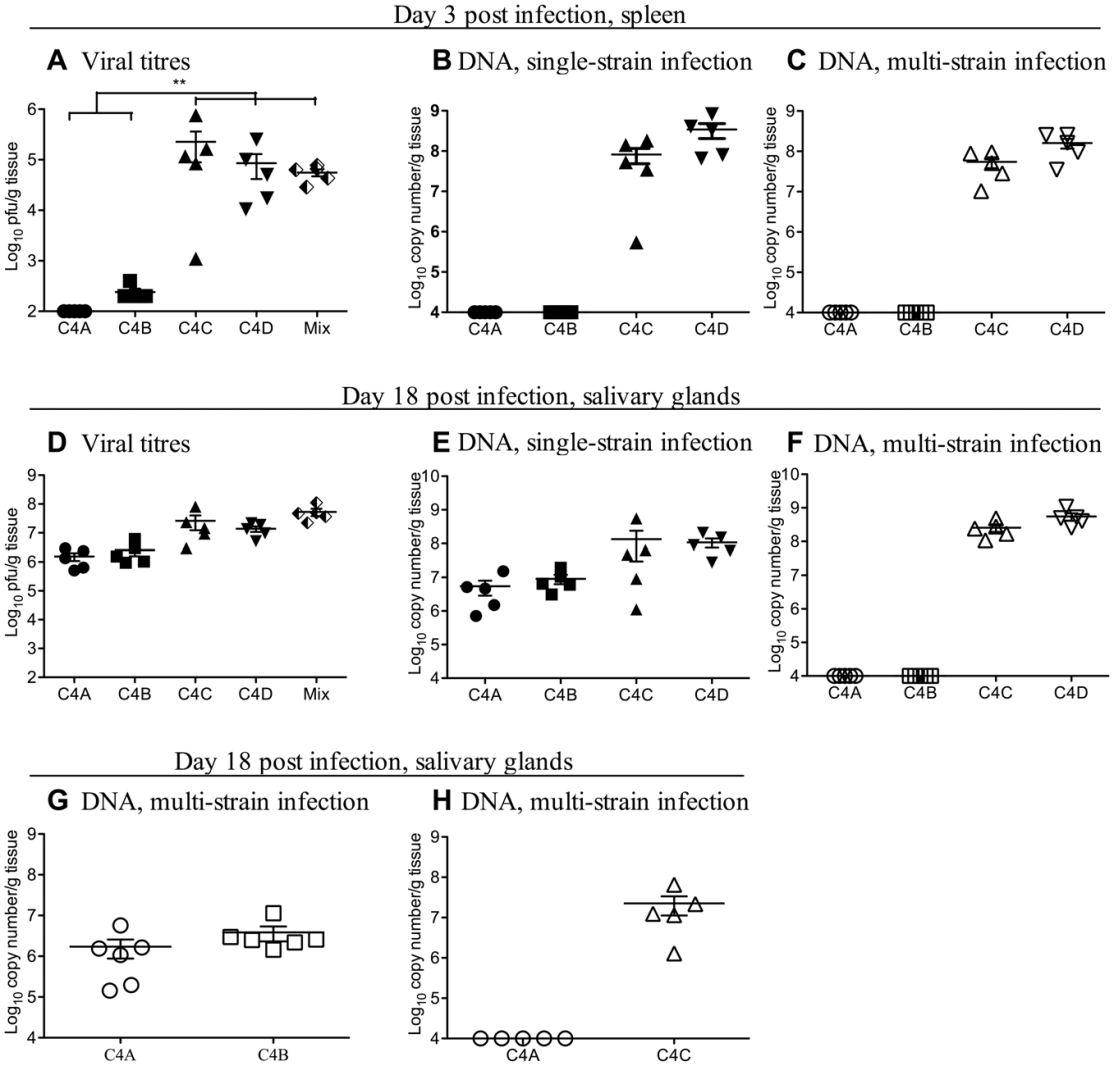
Competition is observed amongst co-infecting strains of MCMV in B6 mice. The outcome of multiple MCMV infection was investigated in B6 mice during acute infection in the spleen (A–C) and persistent infection in the salivary glands (D–H). B6 mice were inoculated i.p. with 1×10^4^ pfu of either a single MCMV strain (closed symbols) or a total of 1×10^4^ pfu of an mixed inoculum of up to four MCMV strains in equal proportions (half filled diamonds - plaque assay or open symbols – strain specific PCR). **A**. Viral titers in the spleens of B6 mice 3 days after single strain (closed symbols) or multi-strain (Mix - half filled diamond) infection. Titers of virus in C4C infected mice were significantly higher (p<0.05) than in all other groups of MCMV infected mice. **B**. Viral DNA levels in the spleen of single strain infected mice from Fig. 1A. **C**. Viral DNA levels in the spleen of multi-strain infected mice from Fig. 1A. **D**. Viral titers in the salivary glands of B6 mice 18 days after single strain (closed symbols) and multi-strain infection (Mix - half filled diamond). **E**. Viral DNA levels in the salivary glands of single strain infected mice from Fig. 1D. Note despite low titers in the spleen, both C4A and C4B replicate in the salivary glands of B6 mice. **F**. Viral DNA levels in the salivary glands of multi-strain infected mice from Fig. 1D. Note during multi-strain infection both C4A and C4B were undetectable by qPCR in the salivary gland. **G**. B6 mice were co-infected with 1×10^4^ pfu of an equal mix of C4A and C4B and the levels of each virus assessed in the salivary glands of mice 18 days later by qPCR. Note both C4A and C4B replicate at this site when co-inoculated together. **H**. B6 mice were co-infected with 1×10^4^ pfu of an equal mix of C4A and C4C, and the levels of each virus assessed in the salivary glands of mice 18 days later by qPCR. Note C4A DNA was undetectable at this site in B6 mice co-infected with both C4A and C4C. The x-axis represents the limit of detection for plaque assays (100 pfu/g of tissue) and the multiplex qPCR (1×10^4^ copy number/g of tissue), n = 5 mice per group.

Female B6 mice were infected with 1×10^4^ pfu of C4A, C4B, C4C or C4D or with an equal mix of all four strains via the peritoneal route. Viral titers were assessed 3 days later in the spleen and the liver. Infectious virus was undetectable in the spleens of C4A inoculated mice 3 days after infection, while titers of virus in C4B infected mice were just above the limit of detection (Figure 1A). In contrast, viral titers were 2.3×10^5^±1.3×10^5^ and 8.6×10^4^±4.3×10^4^ pfu/g tissue in the spleens of C4C and C4D infected mice, respectively (Figure 1A). There was no evidence of enhanced viral replication in the spleens of multi-strain infected B6 mice with titers significantly lower than those seen in C4C infected mice and similar to those seen in C4D infected mice (Figure 1A). The number of viral genomes detected in the spleens of infected mice were equivalent for each MCMV strain irrespective of infection protocol, with no enhancement of C4A or C4B viral DNA levels in multi-strain (Figure 1C) compared to single strain (Figure 1B) infected mice, and no enhancement or reduction in the DNA load of C4C or C4D MCMV strains in multi- compared to single- strain infected mice. Similar data were obtained in the livers of mice three days p.i with no enhancement of viral loads and no apparent interaction between the MCMV strains (data not shown).

Despite low titers of C4A and C4B during acute single strain infection, viral titers in the salivary gland of C4A and C4B singly infected mice at day 18 p.i. were not significantly different to those detected in C4C or C4D infected mice (Figure 1D). Moreover, there was no enhancement of viral titers in the salivary glands of multi-strain infected mice (Figure 1D). Mixed infection did however, have a profound effect on within-host interactions. All four MCMV strains were detectable in the salivary gland by qPCR following single strain infection (Figure 1E). However after co-infection with all four MCMV strains, C4A and C4B were undetectable at this site (Figure 1F). Failure to detect C4A and C4B at this site was not simply due to mixed infection *per se*, as both these strains were able to disseminate to, and replicate within, the salivary gland when administered together (Figure 1G). However, in C4A and C4C co-infected mice, C4A DNA was undetectable in the salivary gland of B6 mice (Figure 1H). Therefore within-host competition was detected between MCMV strains in a virus-strain specific manner.

### Within-host competition is dependent on NK cells

Resistance of B6 mice to particular (m157^Ly49H+^) strains of MCMV is due to the recognition of specific genotypes of the viral m157 glycoprotein by host NK cells via the activation receptor Ly49H [43]–[47]. These m157^Ly49H+^ strains of MCMV replicate poorly in the spleen during acute infection [48], but show prolonged replication in the salivary glands of B6 mice [59]. Strains that avoid Ly49H mediated immunosurveillance (m157^Ly49H−^) replicate well during acute infection but are more rapidly cleared from the salivary glands of B6 mice [48], [59]. Phylogenetic analysis of the *m157* gene sequences from the C4 strains of MCMV suggested a possible mechanism behind viral competition. The m157 genes of C4A and C4B co-segregated with genotypes of m157 known to ligate Ly49H. In contrast, the m157 genes of C4C and C4D co-segregated with m157 genes, shown by others [48], [50], to be incapable of ligating Ly49H (Figure S1).

**Figure 2:**
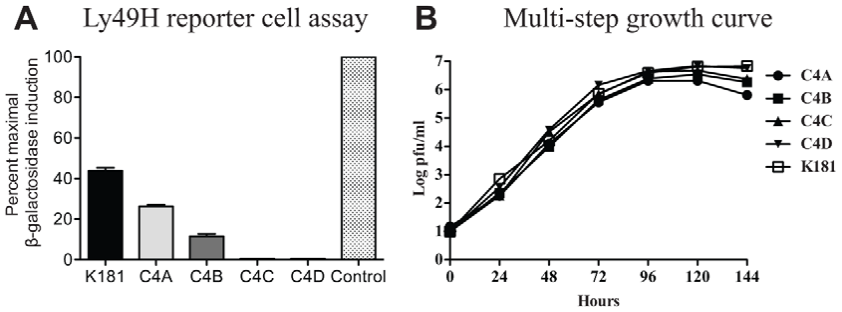
Within-host dominance mirrors the capacity of MCMV strains to ligate Ly49H. **A**. Ly49H reporter cell assay. MEF were infected with K181 (positive control) or the C4 strains of MCMV and used to stimulate the Ly49H reporter cell line BWZ-HD12. The percent maximal β-galactosidase production was quantified relative to the PMA/ionomycin control (stippled bar). Both C4A and C4B stimulated β-galactosidase production by reporter cells, whereas C4C and C4D infected cells failed to stimulate β-galactosidase expression. **B.** Multi-step growth curves for the C4 strains of MCMV. MEF were infected with an MOI of 0.01 and replication rates assessed by plaque assay over a six day period. All viral strains replicated to similar levels in MEF.

To assess the role of Ly49H in within-host competition, the C4 strains of MCMV were tested for the capacity to ligate Ly49H using the reporter cell line, BWZ-HD12 [46], [48]. MEF infected with either C4A or C4B activated the reporter cell line, while those infected with C4C or C4D failed to activate BWZ-HD12 cells (Figure 2A). All strains of MCMV replicated to similar levels in MEF indicating that failure to ligate Ly49H was not due to restricted replication in these cells (Figure 2B). Expression of m157 is low on infected cells and in this study could only be detected on K181 infected MEFs (Figure S2). Failure to detect m157 on the surface of MEF infected with the C4 strains of MCMV may reflect the specificity of the anti-m157 monoclonal antibody (6H121) which was raised against the Smith strain m157 [53]. Smith strain m157 is identical to m157 from K181.

**Figure 3:**
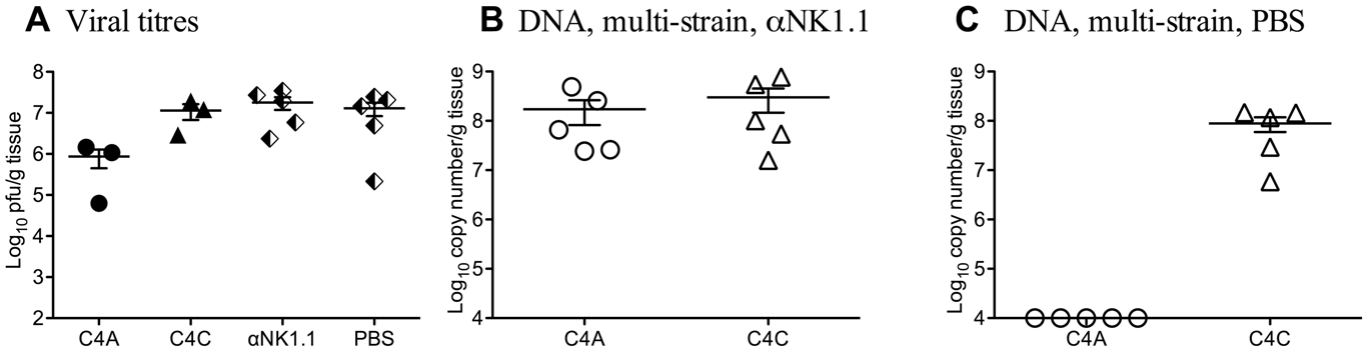
Within-host competition in B6 mice is dependent on NK cells. To assess the role of NK cell on within-host competition, NK cells were depleted from B6 mice by i.p. injection of 150 µg of the monoclonal antibody PK136 at day −1, 0 and then every 5 days until day 15 p.i. On day 0 mice were inoculated i.p. with 1×10^4^ pfu of either a single MCMV strain (closed symbols) or a total of 1×10^4^ pfu of a mixed inoculum of equal proportions of C4A and C4C (half filled diamonds - plaque assay or open symbols – strain specific PCR). **A.** Titers of infectious virus were determined by plaque assay in salivary glands of non-manipulated mice infected with either C4A or C4C (closed symbols), or in PK136 (anti-NK1.1) or PBS treated mice co-infected with C4A and C4C (half filled diamonds). **B**. Viral DNA levels in B6 mice treated with PK136 (anti-NK1.1) and co-infected with C4A and C4C. Note both C4A and C4C DNA were detectable following NK cell depletion. **C**. Viral DNA levels in B6 mice treated with vehicle (PBS) and co-infected with C4A and C4C. Note competition was retained, with C4A DNA undetectable in the salivary glands. The x-axis represents the limit of detection for plaque assays (100 pfu/g of tissue) and the multiplex qPCR (1×10^4^ copy number/g of tissue), n = 5 mice per group.

These data suggested that within-host competition in B6 mice was mediated by NK cells. To test this hypothesis, B6 mice were depleted of NK cells by treatment with anti-NK1.1 (PK136) monoclonal antibody prior to and during co-infection with C4A and C4C (Figure 3A, B). Figure 3A shows infectious viral titers in non-manipulated B6 mice infected with C4A or C4C, and infectious viral titers in mice co-infected with C4A and C4C and treated with either PK136 or PBS. Depletion of NK cells restored the capacity of C4A to replicate in the salivary gland during mixed infection (Figure 3B). C4A was, however, excluded from the salivary gland of co-infected mice treated with vehicle alone (Figure 3C). These data indicate a requirement for NK cells in the establishment of within-host competition. The same results were obtained when the anti-NK1.1 treatment was applied on days −1 and 0 of infection only (data not shown).

### Host Ly49H is required for within-host competition

To determine if Ly49H was required for within-host competition, multi-strain infection was assessed in mice congenic for the natural killer cell complex (NKC). BALB.B6-*Cmv1^r^* mice (Cmv1^r^) possesses the NKC from B6 mice on the BALB/c background [60]. B6.BALB-TC1 mice (TC1) possess the NKC from BALB/c mice on a C57BL/6 background [60]. We hypothesized that within-host competition between C4A and C4C would occur in Ly49H^+^ (B6 and Cmv1^r^) but not in Ly49H^−^ (BALB/c and TC1) mice.

**Figure 4:**
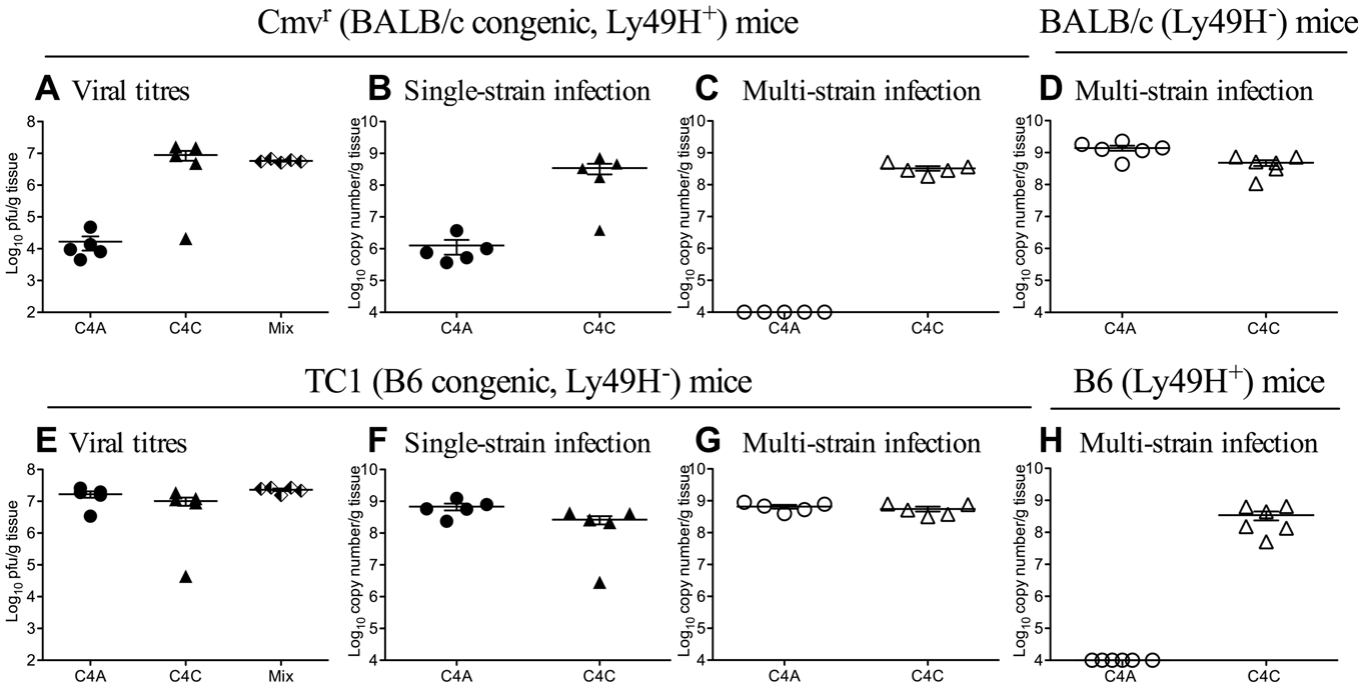
The Cmv1^r^ locus of the natural killer cell complex is linked to within-host competition. Congenic BALB.B6-*Cmv1^r^* (Cmv1^r^) or B6.BALB.TC1 (TC1) mice were inoculated i.p. with 1×10^4^ pfu of either C4A or C4C (closed symbols) or co-infected with a total of 1×10^4^ pfu of C4A and C4C (half filled diamonds - plaque assay or open symbols – strain specific PCR). B6 or BALB/c mice were co-infected with 1×10^4^ pfu of C4A and C4C (open symbols). Viral replication was assessed by plaque assay at day 18 p.i. Strain specific replication was assessed by qPCR. **A**. Total viral loads in the salivary glands of Cmv1^r^ mice infected with C4A, C4C or C4A and C4C (half filled diamonds-Mix). **B**. DNA levels in the salivary glands of single strain infected Cmv1^r^ mice from Fig. 4A. **C**. Viral DNA levels in the salivary glands of multi-strain infected Cmv1^r^ mice from Fig. 4A. **D**. Viral DNA levels in the salivary glands of multi-strain infected BALB/c mice. Note competition was not seen in BALB/c mice, with both C4A and C4C equally well represented in the salivary glands. However, in Ly49H^+^ BALB/c background mice (Cmv1^r^), C4A was excluded from the salivary glands during multi-strain infection. **E**. Total viral loads in the salivary glands of TC1 mice infected with C4A, C4C or C4A and C4C (half filled diamonds-Mix). **F**. DNA levels in the salivary glands of single strain infected TC1 mice from Fig. 4E. **G**. Viral DNA levels in the salivary glands of multi-strain infected TC1 mice from Fig. 4E. **H**. Viral DNA levels in the salivary glands of multi-strain infected B6 mice. Note competition was not seen in Ly49H^−^ B6 background TC1 mice, with both C4A and C4C equally well represented in the salivary glands. However, C4A was undetectable in the salivary glands of co-infected B6 mice. The x-axis represents the limit of detection for plaque assays (100 pfu/g of tissue) and the multiplex qPCR (1×10^4^ copy number/g of tissue), n = 5–6 mice per group.

Both C4A and C4C replicated within the salivary glands of Ly49H^+^ Cmv1^r^ mice during single strain infection (Figure 4A, plaque assay and Figure 4B qPCR). However, C4A was excluded from the salivary glands of these Ly49H^+^ BALB/c background mice during co-infection (Figure 4C). No competition was seen between these two strains of MCMV in Ly49H^−^ BALB/c mice (Figure 4D). Competition was not seen in B6 background Ly49H^−^ TC1 mice during co-infection (Figure 4G), but was seen in Ly49H^+^ B6 mice (Figure 4H). Both C4A and C4C were detectable in the salivary glands of TC1 mice during single strain infection by plaque assay (Figure 4E) and by qPCR (Figure 4F).

**Figure 5:**
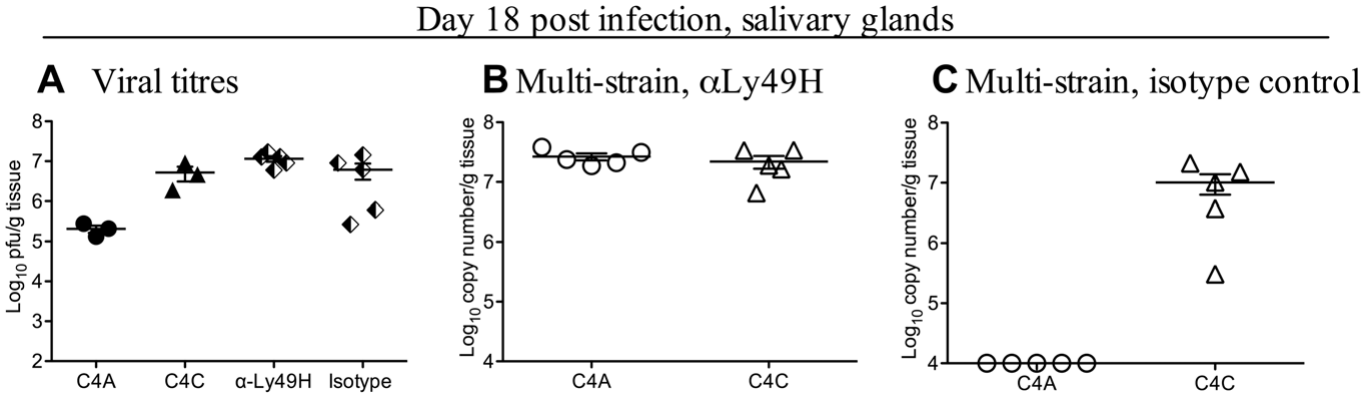
Blocking the NK cell receptor Ly49H eliminates viral competition. B6 mice were treated with 200 µg of the monoclonal antibody 3D10 (anti-Ly49H) or 200 µg of isotype control antibody 9E10 (anti-cMyc) on day −2, 0, 4, 8, 12 and 16 of infection. On day 0 mice were inoculated i.p. with 1×10^4^ pfu of either a single MCMV strain (closed symbols) or a total of 1×10^4^ pfu of a mixed inoculum of C4A and C4C (half filled diamonds - plaque assay or open symbols – strain specific PCR). **A**. Titers of infectious virus were determined by plaque assay in salivary glands of B6 mice infected with either C4A or C4C (closed symbols), or in anti-Ly49H or isotype control treated mice that were also co-infected with C4A and C4C (half filled diamonds). **B**. Viral DNA levels in B6 mice treated with anti-Ly49H and co-infected with C4A and C4C. Note both C4A and C4C DNA were detectable following anti-Ly49H treatment. **C**. Viral DNA levels in B6 mice treated with isotype control mAb and co-infected with C4A and C4C. Note competition was retained in these mice with C4A undetectable in the salivary glands. The x-axis represents the limit of detection for plaque assays (100 pfu/g of tissue) and the multiplex qPCR (1×10^4^ copy number/g of tissue), n = 5 co-infected mice/group, 3 single strain infected mice/group.

To confirm a role for Ly49H, B6 mice were treated with the Ly49H blocking monoclonal antibody 3D10, and co-infected with C4A and C4C (Figure 5A–C). Figure 5A shows infectious viral titers in non-manipulated B6 mice infected with C4A or C4C and infectious viral titers in mice co-infected with C4A and C4C and treated with either monoclonal antibody 3D10 (anti-Ly49H) or 9E10 (anti-cMyc, isotype control). Competition was maintained in isotype control treated mice, with only C4C detectable in the salivary glands (Figure 5C). However, C4A and C4C were detected (at equivalent levels) in the salivary glands of co-infected B6 mice following anti-Ly49H antibody treatment (Figure 5B). Taken together these data confirm an absolute requirement for Ly49H, and therefore NK cells, in the with-host competition between C4A and C4C in B6 mice.

### MCMV strain competition is dependent on virally encoded *m157*

Ly49H mediated competition should be mediated by its cognate ligand, virally encoded m157. The MCMV strain K181 expresses an m157 gene product known to ligate the product of the B6 allele of *Ly49h* [61]. Serial passage of K181 through Ly49H expressing congenic mice results in several different loss-of-function mutations in *m157*. These mutations can be detected in some clones by passage three and are seen in most clones by passage seven [48]. In the study described here, a 104 bp region of *m157* was deleted from K181 to make K181^Δm157^, recreating one of the natural loss–of-function mutations detected by Voigt and colleagues [48]. K181^Δm157^ replicated like wild type virus *in vitro* and failed to activate Ly49H reporter cells (data not shown).

**Figure 6:**
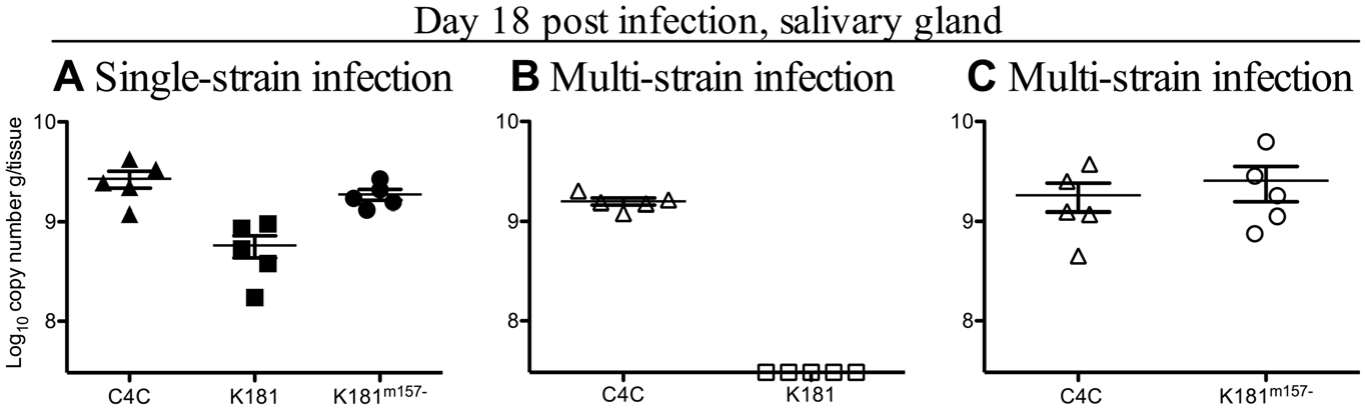
Within-host competition requires m157. A loss of function mutation was generated in the MCMV strain, K181, though the deletion of 104 bp in the m157 gene. B6 mice were inoculated i.p. with 1×10^4^ pfu of either C4C, K181 and or K181^Δm157^ (closed symbols) or inoculated with total of 1×10^4^ pfu of a mixed inoculum of equal proportions of C4C and K181 or C4C and K181^Δm157^ (open symbols). **A** Salivary gland viral DNA levels in single strain infected B6 mice 18 days p.i. **B**. Salivary gland viral DNA levels in C4C and K181 co-infected mice 18 days p.i. **C** Salivary gland viral DNA levels in C4C and K181^Δm157^ co-infected mice 18 days p.i. Note K181 was excluded from the salivary glands when co-infected with C4C. However, deletion of m157 restored the replication of K181 (K181^Δm157^) during multi-strain infection. The x-axis represents the limit of detection for the K181 qPCR (3.09×10^5^ copy number/g of tissue), n = 5 mice/group.

*In vivo*, titers of K181^Δm157^ in the salivary glands of B6 mice 18 days p.i. were higher than those of parental K181 infected B6 mice and similar to those observed in C4C infected mice (Figure 6A). Smith strain MCMV is more virulent following inactivation of m157 [62]. This was also the case for K181. By day three p.i., B6 mice infected with C4C (n = 10) or K181^Δm157^ (n = 10) lost significantly more weight than those infected with K181 (n = 10) (p<0.001). Values are percentage weight loss from day 0 to day 3 p.i. and are mean ± SD for: C4C 10.1±1.9, K181^Δm157^ 12.5±1.9 and K181 (weight gain) +0.6±4.0.

The capacity of m157 to mediate competition was assessed in B6 mice infected with either C4C and K181 or C4C and K181^Δm157^. In B6 mice infected with C4C and K181, only C4C DNA was detected in the salivary gland 18 days p.i. (Figure 6B). These data indicate that competition between m157^Ly49H−^ and m157^Ly49H+^ strains of MCMV is universal and not confined to C4A or C4B. In B6 mice co-infected with C4C and K181^Δm157^, DNA from both strains of MCMV was present in the salivary gland (Figure 6C). These data demonstrate that Ly49H dependent competition was also m157 dependent.

### MCMV strain competition influences viral transmission

The ability of a particular pathogen strain to transmit from host to host ultimately shapes the pathogen population and contributes to overall virulence. Some theoretical models of multi-strain infection predict that within-host competition has the potential to shape the population structure of pathogens by selecting for enhanced virulence (reviewed in [63]). The salivary gland is both an organ of viral persistence as well as a major source of transmission via shedding of virus into the saliva [64]. Within the confines of this model, this means that only the more virulent strains of MCMV (those that avoid Ly49H mediated immunosurveillance) should transmit.

**Figure 7:**
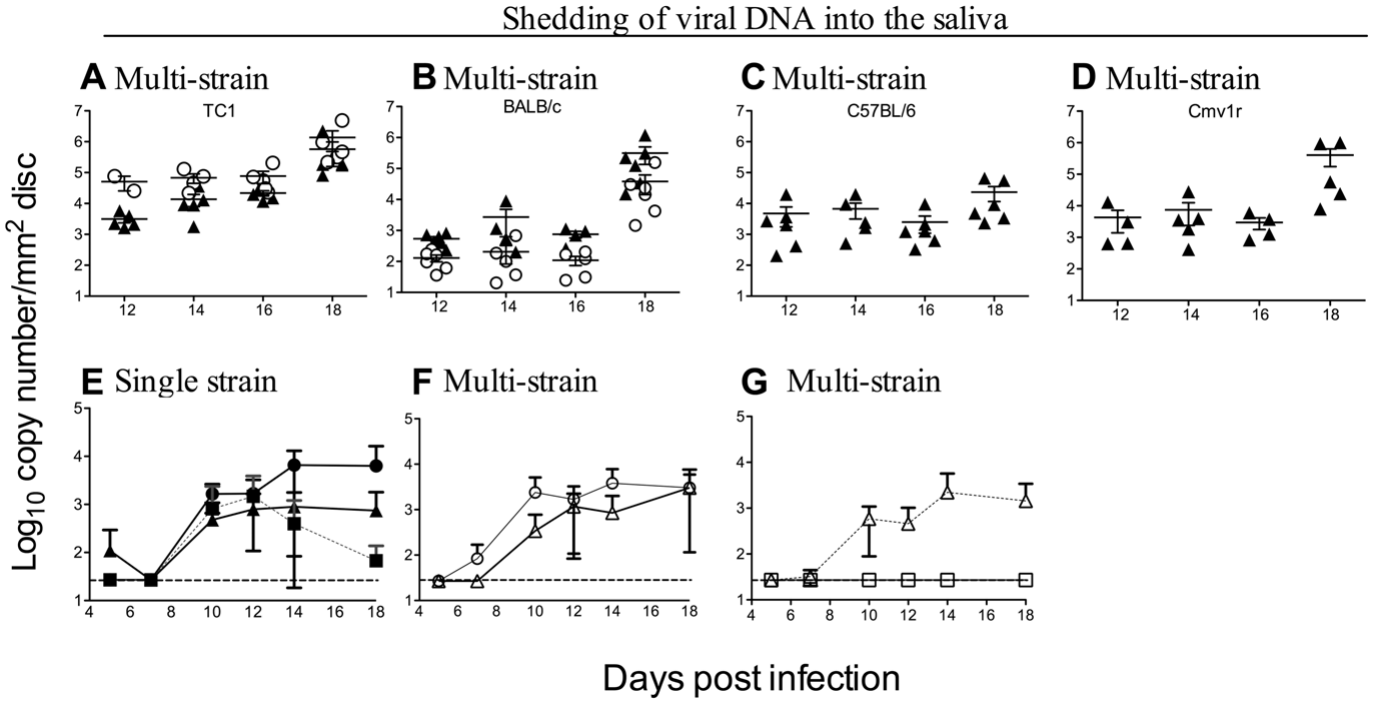
Within-host competition prevents viral shedding into the saliva. Shedding of C4A (open circles) and C4C (filled triangles) was assessed during co-infection (a total of 1×10^4^ pfu of virus given i.p.) in **A**. TC1 mice, **B**. BALB/c mice, **C**. B6 mice and **D**. CMV1^r^ mice. Note, both viruses were present in the saliva of Ly49H^−^ (TC1 and BALB/c) mice, whereas C4A was excluded from the saliva of Ly49H^+^ (B6 and CMV1^r^) mice. The effect of m157 on viral shedding was also assessed. B6 mice were infected with 1×10^4^ pfu of C4C, K181 or K181^Δm157^ or co-infected a total of 1×10^4^ pfu of either C4C and K181^Δm157^ or C4C and K181. Shedding of virus into the saliva was assessed by qPCR **E**. Viral DNA in the saliva of mice infected with C4C (filled triangles), K181 (filled squares) or K181^Δm157^ (filled circles). All three strains of MCMV were shed into the saliva during single strain infection. **F**. Viral DNA in the saliva of mice co-infected with C4C (open triangles) and K181^Δm157^ (open circles). **G**. Viral DNA in the saliva of mice co-infected with C4C (open triangles) and K181 (open squares). Note both C4C and K181^Δm157^ were shed into the saliva of B6 during co-infection, however K181 was excluded from the saliva when co-infected with C4C. Data are mean ± SEM for 5 animals per group. Dotted line indicates limit of detection.

To assess the likely effects of within-host competition on transmission, saliva samples were collected on FTA paper from infected mice (BALB/c, B6, Cmv1^r^ and TC1) from day 12 p.i, and viral DNA quantified by qPCR (Figure 7A–D). Both C4A and C4C were present in nearly equal proportions in saliva samples collected from Ly49H^−^ (TC1 and BALB/c) strains of mice (Figure 7A, B). However, C4A DNA was undetectable in the saliva of Ly49H^+^ (B6 and Cmv1^r^) mice (Figure 7C, D). Similar data were obtained from B6 mice treated with Ly49H blocking antibody, with C4A and C4C DNA present in the saliva of these mice, while only C4C DNA was detected in the saliva of mice treated with isotype control antibody (data not shown).

To further investigate this effect on shedding and therefore transmission, viral DNA levels were assessed in the saliva from mice infected with C4C, K181 or K181^Δm157^ or during co-infection with C4C and K181^Δm157^ or C4C and K181. To determine at what stage of infection competition was first evident, shedding of virus into the saliva was measured from day five p.i. During single strain infection all three viruses were shed into the saliva (Figure 7E). Likewise during co-infection with C4C and K181^Δm157^ both viruses were shed into the saliva (Figure 7F). In contrast, K181 was undetectable in the saliva of B6 mice during co-infection with C4C (Figure 7G). Viral shedding into the saliva was readily detectable by as early as day 10. Competition was well established by this stage suggesting that competition was mediated by a defect in dissemination rather than replication within the salivary glands.

### Within-host competition occurs early, prior to salivary gland dissemination

MCMV replicates during acute infection predominantly in the spleen and liver and disseminates to the salivary gland during a viremic phase approximately 8 days p.i. Within-host competition was apparent in the saliva by day 10 p.i. (Figure 7). Control of viral replication within the salivary glands is not typically associated with NK cell function. Competition was assessed at day three p.i. to determine if NK cells prevented dissemination to the salivary gland or if competition was due to events occurring at this site. K181 was used in these studies as it replicates to higher titres during acute infection in B6 mice than either C4A or C4B.

**Figure 8:**
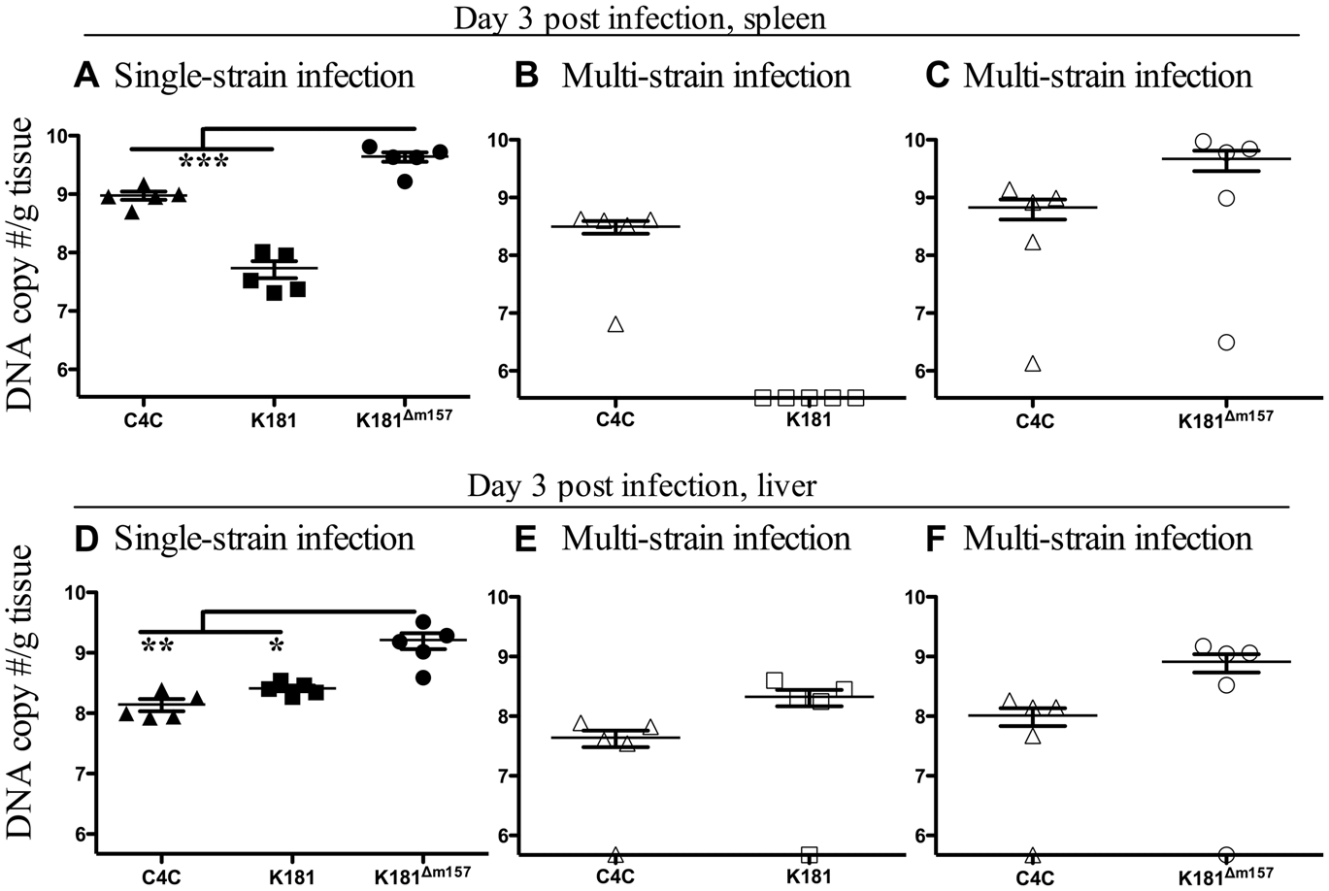
Competition is evident early, before the maturation of acquired immunity. B6 mice were inoculated i.p. with 1×10^4^ pfu of either C4C, K181 or K181^Δm157^ (closed symbols) or a total of 1×10^4^ pfu of a mixed inoculum of equal proportions of with C4C and K181 or C4C and K181^Δm157^ (open symbols). **A**. Viral DNA levels in the spleens of B6 mice three days after single strain infection. **B**. Viral DNA levels in the spleens of mice co-infected with C4C and K181. **C**. Viral DNA levels in the spleens of mice co-infected with C4C and K181^Δm157^ **D**. Viral DNA levels in the livers of B6 mice three days after single strain infection. **E**. Viral DNA levels in the livers of mice co-infected with C4C and K181. **F**. Viral DNA levels in the spleen of mice co-infected with C4C and K181^Δm157^. Note competition was evident in the spleen (B) but not in the liver (E). Data are mean ± SEM for 5 animals per group. Dotted line indicates limit of detection. DNA levels in mice following single strain infection were compared by one-way Anova, asterisks indicate level of significance after Tukey's post hoc analysis (*** P<0.001, ** P<0.01, * P<0.05).

B6 mice were infected with C4C, K181, K181^Δm157^ or C4C and K181 or C4C and K181^Δm157^. Surprisingly competition was apparent in the spleen of B6 mice by day three p.i. While DNA levels of K181^Δm157^ were significantly higher in the spleens of B6 mice than C4C during single strain infection (Figure 8A), DNA from both viruses was detectable in this organ during co-infection (Figure 8C). Therefore enhanced replication, K181^Δm157^ compared to C4C, was not sufficient to promote competition between strains of MCMV. In contrast, only C4C DNA was detectable in the spleens of B6 mice co-infected with C4C and K181 (Figure 8B). K181 DNA was however present in the spleen of B6 mice infected with K181 alone (Figure 8A).

Viral titres in the spleen are more sensitive to inoculum size than in the salivary glands, and the absence of K181 during multi-strain infection could simply reflect the two-fold reduction in K181 inoculated into multi-strain infected mice. However, K181 DNA levels in the spleens of B6 mice inoculated with 5×10^3^ pfu (2.4.×10^7^±1.3×10^7^ g/tissue) were not significantly different to those in mice given an inoculum of 1×10^4^ pfu of K181 (5.4×10^7^±1.8×10^7^ g/tissue). Moreover similar data were obtained in a repeat experiment where single strain infected mice were inoculated with 5×10^3^ pfu of either C4C, K181 or K181^Δm157^ and multi-strain infected mice were inoculated with a total of 1×10^4^ pfu of an equal combination of either C4C and K181 or C4C and K181^Δm157^. In this instance single and multi-strain infected mice received identical inoculums of each individual MCMV strain. Despite this, competition was once again seen in C4C and K181 co-infected mice but not in C4C and K181^Δm157^ co-infected mice (data not shown). Hence competition is apparent in the spleens of multi-strain infected mice before the maturation of acquired immunity and prior to dissemination to the salivary glands.

In contrast to the spleen, competition was not seen in the liver of B6 mice (Figure 8D–F). In this organ, both C4C and K181 were present in co-infected mice (Figure 8E). Likewise both C4C and K181^Δm157^ were present during multi-strain infection in the liver (Figure 8F). Similar data were seen in a repeat experiment (data not shown). Therefore, patterns of competition in the spleen and liver exactly mirror the Ly49H mediated control of MCMV infection in B6 mice, which is expressed in the spleen but not the liver [57]. These data reinforce the central role of NK cells in mediating competition in B6 mice.

## Discussion

The Ly49H/m157 axis has provided many insights into the interaction of mice with MCMV and also with more fundamental aspects of immunology such as the potential of NK cells to exhibit memory responses [65]. Here we have used this axis to probe the outcome of multi-strain infection. In B6 mice, multiple-infection with MCMV resulted in profound competition in which m157^Ly49H+^ strains of MCMV failed to disseminate to, or replicate within the salivary glands. Competition was not simply a reflection of multi-strain infection as no competition was seen in mice infected with only C4A and C4B, two m157^Ly49H+^ strains of MCMV. Substitution of C4B with C4C (m157^L49H−^) resulted in the establishment of competition, indicating that the genetics of the co-infecting viral strains was important. NK cell depletion using PK136 (anti-NK1.1) suggested a role for NK cells in mediating within-host competition, however NK1.1 is also expressed on natural killer T cells. NKC congenic mice were used to demonstrate that competition among MCMV strains occurred only in mice that naturally expressed or were congenic for Ly49H. Antibody blocking studies supported an absolute requirement for Ly49H, an NK cell specific receptor [66]. Deletion of m157 from K181, an m157^Ly49H+^ strain of MCMV, removed competition during co-infection with the m157^Ly49H−^ strain C4C, indicating a requirement for m157 binding of Ly49H. Finally, competition was rapid, prior to the maturation of acquired immunity, and exactly mirrored the tissue distribution of Ly49H immunosurveillance, with competition seen in the spleen but not the liver [57].

The rapidity at which m157^Ly49H+^ strains of MCMV were excluded from the salivary glands suggests that NK cells mediate this competition by preventing the dissemination of MCMV to the salivary glands. This hypothesis is consistent with the rapid competition observed in the spleen but appears at odds with the lack of competition seen in the liver. Presumably virus from the liver (and perhaps other organs) could serve as a reservoir for viral dissemination to the salivary gland. However, the origin of MCMV colonizing the salivary glands is unclear. The established paradigm is that infecting virus replicates at the site of entry and then later in secondary organs before final dissemination to the target organ [67]. For MCMV this is in the peritoneal cavity, when injected via this route, which leads to a primary viremia and dissemination of the virus to the spleen and liver. This is then followed by a secondary viremia and dissemination of the virus to the salivary gland from which it is transmitted to a new host [68]. However this picture of CMV dissemination has recently been challenged. Sacher and colleagues (2008) demonstrated that hepatocytes, the major producer of virus in the liver, were not the source of MCMV in the salivary glands [69]. Whilst the origins of virus in the salivary glands is therefore unclear, the data presented here support the proposition that NK cells mediate entirely the competition seen in this model. Moreover, they prevent the dissemination of MCMV to the salivary gland, and in doing so prevent transmission of m157^Ly49H+^ strains of MCMV during multi-strain infection.

The “apparent competition” (competition mediated by host immunity) seen in this model has been predicted to be important in shaping interactions amongst co-infecting pathogen strains [1], [63]. Apparent competition between strains of pathogen can be mediated by adaptive immunity, such as that seen between strains of *Plasmodium chabaudi* [70] or by innate immunity, such as that seen between encapsulated and non-capsulated strains of *Streptococcus pneumonia* during co-infection with *Haemeopilus influenzae* [71]. Here we show for the first time that NK cells, due to strain specific recognition, can mediate within-host competition. Moreover we identify the exact molecular mechanism that leads to within host competition, that being the Ly49H/m157 axis.

Transmission of pathogens from one host to another is ultimately the most important aspect of pathogen of behavior. Here we have demonstrated that competitive interactions amongst co-infecting strains of MCMV have the capacity to influence which strains are transmitted. For CMV, the salivary gland is an important organ for viral persistence and transmission [64]. In B6 mice competition between m157^Ly49H+^ and m157^Ly49H−^ MCMV strains resulted in the exclusion of m157^Ly49H+^ strains from the saliva. Avoidance of Ly49H mediated immunosurveillance leads to enhanced virulence of MCMV in B6 mice as measured by higher viral titers and enhanced morbidity (data described herein and [62]). Within-host competition therefore leads to selective shedding of the more virulent strain of MCMV. These data fit theoretical models which predict that within-host competition will drive the evolution of virulence by selecting for transmission of more virulent pathogen strains (reviewed in [1]). Virulence in these models is often ascribed to pathogen replication rates. Here virulence is linked to avoidance of a dominant host control mechanism.

Mutation of m157 following passage in Ly49H^+^ mice is a potential mechanism for the evolution of this viral gene [48], [72]. Here we show another potential mechanism for the loss of specific viral genotypes from the population that does not require mutation and loss of function. Since multi-strain MCMV infection is common in free-living mice [41], [42] this may be a powerful mechanism for the selection of non-Ly49H binding genotypes of m157. In populations of mice where *Ly49h* and multi-strain infection is common these data would predict rapid loss of m157^Ly49H+^ strains of MCMV from the population. Under these circumstances there may be a reduced role for mutational inactivation of m157. This would permit the retention of m157 function and allow for continued targeting of inhibitory Ly49 molecules [47], [50] by MCMV. When Ly49H mediated selection of m157 mutations following serial viral passage have been demonstrated, the vast majority of these mutations are frame shift mutations, premature stop mutations or deletions in the hydrophobic tails [48], [72]. These types of mutations are not apparent in the m157 gene sequences from the 36 different MCMV strains so far deposited in DNA databases. Importantly, this suggests that mutation of m157 is not generally required for the removal of m157^Ly49H+^ genotypes from a population, and that multi-strain infection can lead to rapid (within a single passage) loss of these genotypes without inactivation of the m157 gene.

The capacity of NK cells to shape the population dynamics of CMV infection may not be limited to the effects of Ly49H and m157, as NK cell targeting of specific viral proteins is not confined to the Ly49H/m157 axis. In BALB.K mice resistance to MCMV is due to Ly49L^+^ NK cells, and these cells proliferate in response to MCMV infection as is seen for Ly49H^+^ NK cells in B6 mice [73]. In BALB.K mice, Ly49L^+^ NK cells specifically respond to MCMV encoded m04 [73]. Like m157, m04 varies between strains of MCMV [49], [74], [75] allowing for the possibility that genes other than m157, and NK cells other than those expressing Ly49H, could shape within-host interactions between strains of MCMV. Humans also encode a series of polymorphic inhibitory and activating NK cell receptors called killer cell immunoglobulin-like receptors (KIR) which are functionally analogous to the mouse Ly49 family [76]. While activating receptors recognizing HCMV encoded ligands have not been demonstrated, HCMV seropositive individuals have higher levels of circulating CD94/NKG2C^+^ NK cells [77]–[79] which expand rapidly after acute HCMV infection or reactivation in transplant recipients [80], [81]. Furthermore, like MCMV encoded m04 and m157, HCMV expressed targets of NK cells such as UL18 [77], [78] also vary between viral strains [79]. Therefore it is possible that NK cells will also be able to mediate strain specific recognition of HCMV which would provide the basis for NK cell mediated within host competition during multi-strain HCMV infection. Interestingly, strains of HCMV are known to vary in prevalence during longitudinal studies in infected individuals [23] suggesting stochastic reactivation from latency - as suggested by the study authors - or perhaps competition between the strains. The studies described here provide a paradigm for testing the roles of NK cells in shaping competition between strains of pathogens where multi-strain infection is common.

The mechanisms through which Ly49H mediated immunosurveillance drives such profound competition and control of m157^Ly49H+^ strains of MCMV only during multi-strain infection remain unclear. However it is possible that multi-strain infection alters the amplitude or duration of NK cell responses. Perhaps, during multi-strain infection type I interferons or cytokines such as IL-12 and IL-15 are maintained at higher levels in response to the continued presence of m157^Ly49H−^ strains of MCMV. This could result in either an enhanced expansion of the Ly49H^+^ NK cell pool or a delay in the contraction phase [80]. Alternatively, prolonged or enhanced expression of these cytokines may modulate either the recruitment of naïve NK cells [81] into the activated pool of NK cells or moderate the “anergy” seen in Ly49H^+^ NK cells following repeated stimulation through this receptor [82], [83]. The rapidity of the competition, as noted in the spleen, suggests that alterations to NK cell responses must be rapid. However, it is likely that altered NK cell responses may also be prolonged and required for the prevention of dissemination from other organs not assessed in this study.

In summary our data demonstrate that NK cells have the strain specific recognition capacity to meditate within-host competition between strains of MCMV. This competition has the capacity to shape the dynamics of viral shedding, and to select for the more virulent strains of MCMV that avoid Ly49H mediated immunosurveillance. This within-host competition provides a potential mechanism for the rapid removal of m157^Ly49H+^ binding strains of MCMV from a population without the need for mutational inactivation of the m157 gene.

## Supporting Information

Figure S1 **Phylogenetic analysis of published m157 sequences.** Sequences of m157 were extracted from DNA databases or produced in this study and used to construct a maximum likelihood phylogeny. Shown in red are m157 genotypes that have been shown to encode products capable of ligating Ly49H. In green are genotypes that produce products that do not ligate Ly49H. Genotypes in black have not been tested. Functional data was obtained from publications 48 and 50 and data described herein.
(EPS)

Figure S2 **Surface expression of m157 on infected MEF.** MEF were infected with high MOI (>10) and stained for surface expression of m157 using the monoclonal antibody 6H121. 6H121 was raised against the Smith/K181 variant of m157. Uninfected cells (grey shading) were used as controls. Note m157 was only detectable on the surface of MEF infected with K181, solid black line – last histogram. Surface expression of m157 was not detectable on C4A, C4B, C4C or C4D (grey lines – order is front to back) infected cells.
(EPS)
